# Supplementary material for: The Effect of Rare Earths on the Response of Photo UV-Activate ZnO Gas Sensors
Source: Sensors (Basel). 2022 Oct 25;22(21):8150. doi: 10.3390/s22218150 (PMC9658068; doi:10.3390/s22218150)
Supplement: Supplementary file 1 [file sensors-22-08150-s001.zip › sensors-1985882-supplementary-v3.pdf]

Detections were carried out during long periods of operation in some cases higher than 11 hours, to establish the sensor stability and the sensor response repeatability. For this purpose, successive detections of one of the target gases were carried out, varying its concentration and registering its resistance value every 30 seconds. The concentrations were modified alternatively (increasing/decreasing/increasing) and the sensors were illuminated by UV light during all the experiment. The sensors were stable, the detection processes repetitive and the sensor responses were similar (same order of magnitude) for the same detected concentration as it can be seen in below Figure S1.

Regarding the sensor response in the long term, sensors were continuously tested for about two months. Some responses to tested gases in different periods of time are shown in the following tables (Tables S1 and S2). The sensors show similar responses.

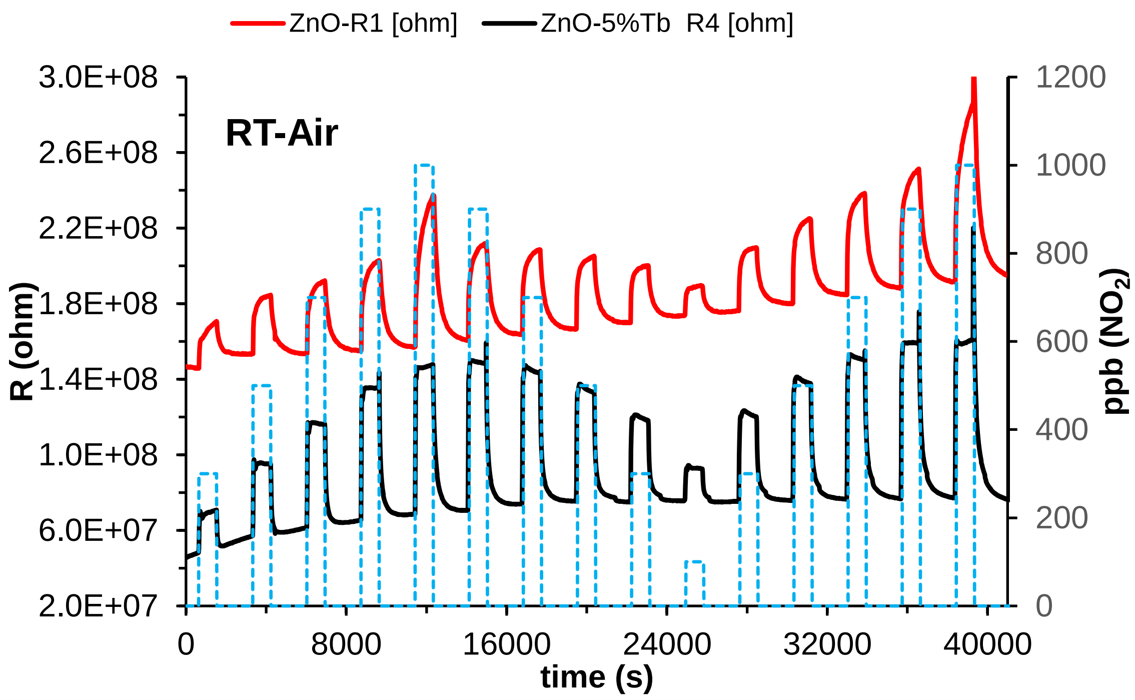

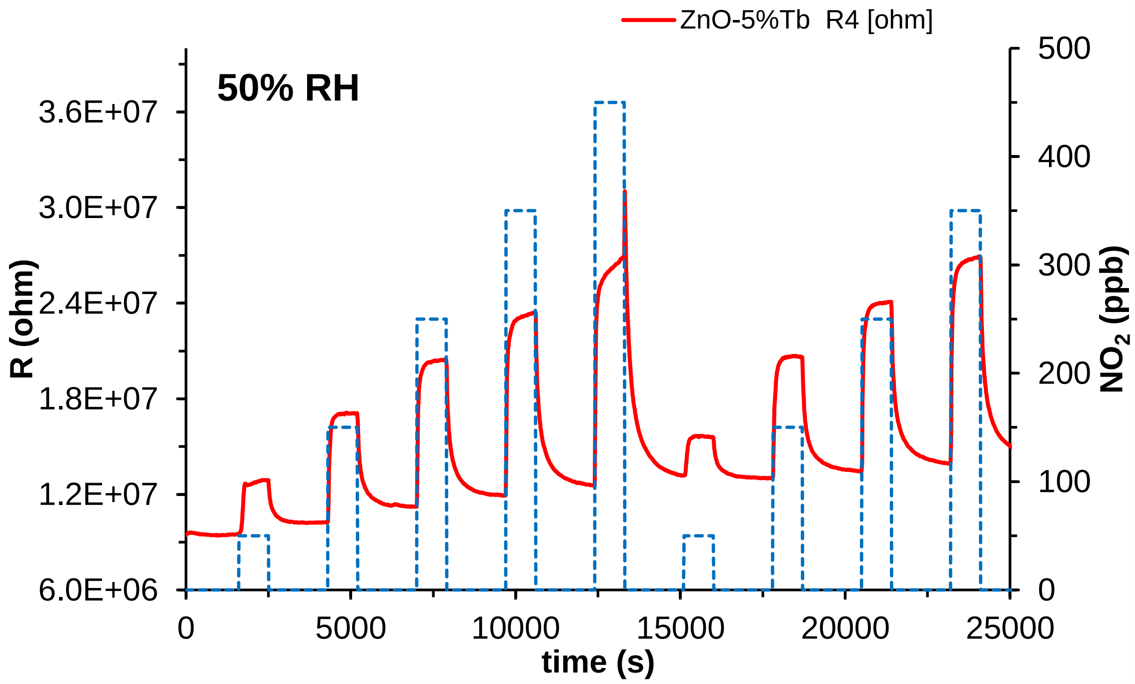

Figure S1. Response curves to NO2 corresponding to some sensors tested during a long time period of operation.

Table S1. Initial sensor response.

Sensor	NO_2 (ppb)	Response (R_{NO2}-R_a/R_a*100)
ZnO-5%Tb	300	47.1
	500	66.6
	700	89.15
	1000	115.2

Table S2. Sensor response after a month.

Sensor	NO_2 (ppb)	Response (R_{NO2}-R_a/R_a*100)
ZnO-5%Tb	100	21.3
	300	51.55
	500	64.53
	700	93.12
	900	103.6
